# Polymorphic variants of the genes for enzymes of the antioxidant system, apoptosis and inflammation as potential predictors of myocardial infarction

**DOI:** 10.18699/vjgb-24-87

**Published:** 2024-11

**Authors:** T.R. Nasibullin, V.V. Erdman, Y.R. Timasheva, I.A. Tuktarova, A.A. Petinseva, G.F. Korytina

**Affiliations:** Institute of Biochemistry and Genetics – Subdivision of the Ufa Federal Research Centre of the Russian Academy of Sciences, Ufa, Russia; Institute of Biochemistry and Genetics – Subdivision of the Ufa Federal Research Centre of the Russian Academy of Sciences, Ufa, Russia; Institute of Biochemistry and Genetics – Subdivision of the Ufa Federal Research Centre of the Russian Academy of Sciences, Ufa, Russia; Institute of Biochemistry and Genetics – Subdivision of the Ufa Federal Research Centre of the Russian Academy of Sciences, Ufa, Russia; Institute of Biochemistry and Genetics – Subdivision of the Ufa Federal Research Centre of the Russian Academy of Sciences, Ufa, Russia Ufa University of Science and Technology, Ufa, Russia; Institute of Biochemistry and Genetics – Subdivision of the Ufa Federal Research Centre of the Russian Academy of Sciences, Ufa, Russia

**Keywords:** myocardial infarction, oxidative stress, apoptosis, inflammation, инфаркт миокарда, окислительный стресс, апоптоз, воспаление

## Abstract

Myocardial infarction (MI) is a multifactorial polygenic disease that develops as a result of a complex interaction of numerous genetic factors and the external environment. Accordingly, the contribution of each of them separately is usually not large and may significantly depend on the state of other accompanying factors. The purpose of the study was to search for informative predictors of MI risk based on polygenic analysis of polymorphic variants of (1) the antioxidant defense enzyme genes PON1 (rs662), PON2 (rs7493), CAT (rs1001179), MSRA (rs10098474) and GSTP1 (rs1695); (2) the apoptosis genes CASP8 (rs3834129), TP53 (rs1042522) and BCL2 (rs12454712); and (3) the inflammation genes CRP (rs1205), CX3CR1 (rs3732378), IL6 (rs1800795) and CCL2 (rs1024611). 591 DNA samples were used in the study (280 patients with the onset at 30 to 60 years, with an average age of 46.02 ± 6.17, and 311 control subjects aged 30 to 62, with an average age of 44.65 ± 7.07). All the participants were male and Tatars by ethnicity. The logistic regression analysis with various models demonstrated associations with MI of polymorphic variants of the genes CX3CR1 (rs3732378) (overdominant model – G/G + A/A vs A/G P = 0.0002, OR = 1.9), MSRA (rs10098474) (dominant model – T/T vs T/C + C/C P = 0.015, OR = 1.51), CCL2 (rs1024611) (recessive model – P = 0.0007 – A/A + A/G vs G/G OR = 2.63), BCL2 (rs12454712) (log-additive model – *C allele, P = 0.005, OR = 1.38). Using the Monte Carlo method and Markov chains (APSampler), combinations of alleles/genotypes of the studied polymorphic loci associated with a high risk of MI were obtained, which, in addition to those identified during single-locus analysis, contained polymorphic variants of the genes CASP8, TP53, CAT, PON2, CRP, IL6, GSTP1. Among the combinations obtained, a pairwise analysis of possible non-linear interactions between the identified combinations of alleles/genotypes was carried out, which showed synergistic interactions of the polymorphic variants CX3CR1*A/G and CASP8*I/I, MSRA*C and CRP*C, CAT*C/T and MSRA*C, CAT*C/T and CX3CR1*A contributing to the development of MI. Based on the results obtained using multivariate logistic regression analysis, a predictive model was built to assess the risk of developing MI, the predictive ability of which reached the value AUC = 0.71 (AUC – area under the curve in ROC analysis).

## Abstract (ru)

Инфаркт миокарда (ИМ) – многофакторное полигенное заболевание, развивающееся в результате сложного взаимодействия многочисленных генетических факторов и внешней среды. Соответственно, вклад каждого из них по отдельности, как правило, невелик и может существенно зависеть от состояния других сопутствующих факторов. Цель исследования – поиск информативных предикторов развития ИМ на основе полигенного анализа полиморфных вариантов генов ферментов антиоксидантной защиты – PON1 (rs662), PON2 (rs7493), CAT (rs1001179), MSRA (rs10098474), GSTP1 (rs1695); апоптоза – CASP8 (rs3834129), TP53 (rs1042522), BCL2 (rs12454712); воспаления CRP (rs1205), CX3CR1 (rs3732378), IL6 (rs1800795), CCL2 (rs1024611). В работе использованы образцы: 591 – ДНК (280 больных, перенесших ИМ в возрасте от 30 до 60 лет, средний возраст 46.02 ± 6.17; 311 – контроль, возраст от 30 до 62 лет, средний возраст 44.65 ± 7.07). Все участники исследования – мужчины, татары по этнической принадлежности. С помощью логистического регрессионного анализа с учетом различных моделей выявлены ассоциации с ИМ полиморфных вариантов генов CX3CR1 (rs3732378) (сверхдоминантная модель – G/G+A/A vs A/G Р = 0.0002, OR = 1.9), MSRA (rs10098474) (доминантная модель – T/T vs T/C+C/C Р = 0.015, OR = 1.51), CCL2 (rs1024611) (рецессивная модель – P = 0.0007 – A/A+A/G vs G/G OR = 2.63), BCL2 (rs12454712) (лог-аддитивная модель – аллель *C, P = 0.005, OR = 1.38). C применением метода Монте-Карло и цепей Маркова (APSampler) получены сочетания аллелей/ генотипов изученных полиморфных локусов, ассоциированных с высоким риском ИМ, в составе которых, помимо обнаруженных в ходе анализа ассоциаций ИМ и отдельных полиморфных вариантов, присутствуют полиморфные варианты генов CASP8, TP53, CAT, PON2, CRP, IL6, GSTP1. Среди этих сочетаний проведен попарный анализ возможного нелинейного взаимодействия между выявленными комбинациями аллелей/генотипов, который показал синергетические взаимодействия полиморфных вариантов CX3CR1*A/G и CASP8*I/I, MSRA*C и CRP*C, CAT*С/T и MSRA*C, CAT*C/T и CX3CR1*A, способствующие развитию ИМ. На основе полученных результатов с использованием многофакторного логистического регрессионного анализа построена предиктивная модель для оценки риска развития ИМ, предсказательная способность которой достигла значения AUC = 0.71 (AUC (area under curve) – площадь под кривой при ROC-анализе).

## Introduction

Myocardial infarction (MI), the most severe clinical variant of coronary heart disease (CHD), significantly reduces life expectancy and quality of life (Shalnova et al., 2022; Sabgayda et al, 2023). In this regard, analysis of its development factors is a crucial task for its prevention. An aggravated family history is one of the main independent risk factors, confirmed by large-scale prospective studies (Colditz et al., 1991; Assmann et al., 2002).

Currently, the molecular genetic basis of hereditary predisposition to MI is actively studied. Genome-wide association studies (GWAS) helped identify a significant number of polymorphic variants associated with CHD in general and MI in particular. At the same time, the obtained results have weak reproducibility; in addition, despite significant advances in the search for genetic variants associated with the pathology under study being made, they do not yield progress in disease prediction.

MI is a multifactorial polygenic disease caused by numerous complexly interacting genetic and environmental factors; the role of individual factors is usually small; moreover, it varies significantly depending on the environment (Domingo et al., 2019). In this regard, a promising direction lies in analyzing associations of polymorphic variant combinations with the studied polygenic trait. At the same time, since increasing the number of elements that make up a combination exponentially increases possible combinations and, as a consequence, decreases the frequency of their occurrence, it seems more rational to limit the number of variables based on already known data on the pathogenesis of the disease, or to include in the analysis polymorphic variants that, according to GWAS, are associated with the studied phenotype.

As known, reactive oxygen species formed during various oxidation-reduction reactions can have a damaging effect on cellular structures and initiate the oxidation of lipids, proteins, and nucleic acids (Batty et al., 2022). Depending on the strength of the effect, they can initiate either inflammation or apoptosis which play a significant role in atherosclerosis development.

Based on the above, this study aims to comprehensively analyze polymorphic variants of the genes of antioxidant defense, inflammation, and apoptosis enzymes (Table 1) as potential predictors of the risk of MI.

**Table 1.**
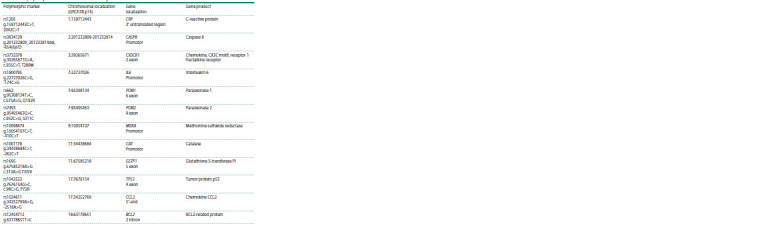
The polymorphic variants included in the study and their location

## Materials and methods

The study material was DNA samples of unrelated patients with onset of large-focal MI at the age of 30 to 60 years (N = 280, the mean age was 46.02 ± 6.17). All the patients were treated at the Republic Center of Cardiology, Ufa

MI was diagnosed on the basis of the 2012 AHA/ESC guidelines using contemporary instrumental and biochemical methods, including 12- lead ECG, echocardiography, radiography of the thoracic organs, clinical and biochemical blood tests, and assessment of myocardial necrosis markers and lipid spectrum. Patients with endocrine pathology and other concomitant severe chronic diseases were excluded. The control group included unrelated individuals at the age of 30 to 62 years (N = 311, the mean age was 44.65 ± 7.08) without clinical signs of cardiovascular pathology. All participants were men belonging to the Tatar ethnic origin, living in Ufa, Republic of Bashkortostan. The study was approved by the Ethics Committee of the Institute of Biochemistry and Genetics (Protocol No. 14 dated 22.12.2010). All participants gave their informed consent.

DNA was isolated by phenol-chloroform extraction. The polymorphic variant rs3834129 (CASP8) was genotyped using PCR followed by separation of the obtained fragments in a 7 % polyacrylamide gel. The remaining polymorphic markers were analyzed with allele-specific PCR, followed by analysis of the fragments on a 2 % agarose gel. The selection of primers for the PCR was carried out using the National Center for Biotechnology Information (NCBI) database (https://www.ncbi.nlm.nih.gov/snp/) and an online tool (https://www.ncbi.nlm.nih.gov/tools/primer-blast/). The primer sequences and expected fragment sizes are presented in Table 2.

**Table 2.**
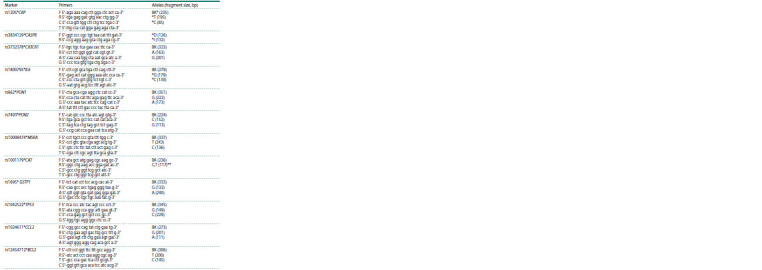
Primer sequences and sizes of amplified fragments *IC – Internal control. ** – First, testing was carried out for the presence of a rare allele *T, then, with a positive test, the sample was tested for the presence of the allele *C.

Statistical analysis of single polymorphic variants was carried out using the tools of the R programming language and the SNPassoc package. The Fisher exact test was used to analyze the deviation of the obtained genotype frequencies from the Hardy–Weinberg equilibrium. When searching for associations with the disease, a logistic regression analysis was used, taking into account five possible inheritance models (codominant, dominant, recessive, overdominant and additive). The best model was chosen according to the Akaike information criterion. The polymorphic marker was considered to be associated with the trait at P < 0.05.

The search for combinations of alleles of genotypes associated with the disease was carried out using the Monte Carlo method and Markov chains using the APSampler software (Favorov et al., 2005). The selection criteria for the identified combinations were P <0.05 after the Benjamin–Hochberg (FDR) procedure and OR < 0.3 (OR is odds ratio) for protective markers or OR > 3 for high-risk markers.

To identify possible nonlinear interaction (synergy) between two elements of the found combinations, the SF (Synergy Factor) indicator was calculated (Cortina-Borja al., 2009). The synergy factor was considered significant if at P < 0.05 and the value of 95 % CI for SF did not cross 1. When constructing predictive models (using SPSS v. 22), the method of multifactorial logistic regression with step-by-step inclusion of variables was used, as which the studied polymorphic variants were selected taking into account the selected optimal model and paired combinations with a significant SF indicator.

## Results

Table 3 shows the results of analyzing the genotype frequency distribution of the studied polymorphic variants. In the control group, all the obtained genotype frequency distributions of the studied loci correspond to the Hardy–Weinberg equilibrium distribution

**Table 3.**
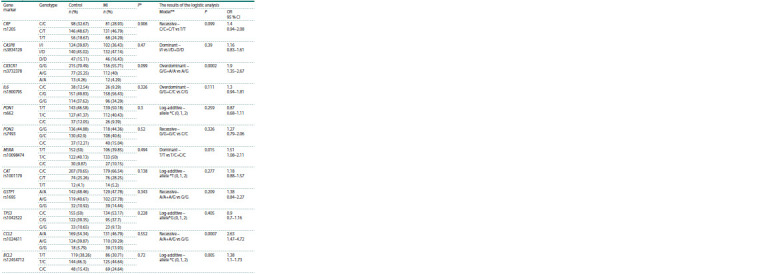
Distribution of genotype frequencies according to the studied polymorphic variants and the results of the analysis of associations with myocardial infarction * The exact test for compliance with the Hardy-Weinberg equilibrium for the control group. ** The model was selected based on the results of the Akaike information criterion.

The analysis of polymorphic loci associations with MI revealed statistically significant results for polymorphic variants of the CX3CR1 (rs3834129), MSRA (rs10098474), CCL2 (rs1024611), and BCL2 (rs12454712) genes. Notably, after introducing the Benjamini–Hochberg correction (multiple comparisons), only the results for the CCL2 and CX3CR1 genes remained significant.

The APSampler software, which employs the dynamic Monte Carlo method, analyzed possible combinations of the studied polygenic variants associated with a high risk of MI. Combinations with higher OR and P values than those of the components of these combinations were identified (Table 4). In this case, the combinations include not only the CX3CR1, MSRA, CCL2, and BCL2 gene variants obtained during the analysis of individual loci but also the CRP, CASP8, PON2, CAT, IL6, GSTP1, and TP53 gene variants.

**Table 4.**
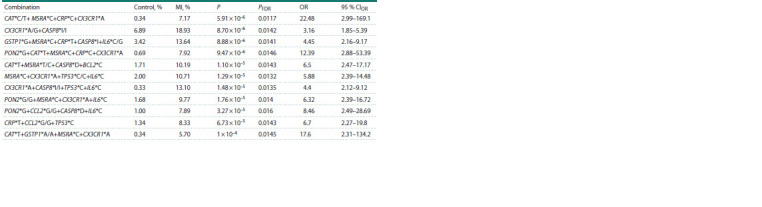
Combinations of alleles/genotypes of the studied polymorphic variants associated with the risk of myocardial infarction

To determine possible non-linear interactions in the identified combinations, the SF factor between all possible pairs of loci included in the obtained combinations was calculated. As a result of the analysis, five statistically significant pairs were obtained: CAT*T+MSRA*C (SF = 2.57, 95 % CISF 1.23–5.4, Z = 2.50, P = 0.01), CAT*C/T+CX3CR1*A (SF = 2.45, 95 % CISF 1.08–5.56, Z = 2.15, P = 0.03), CX3CR1*A/G+CASP8*I/I (SF = 4.71,95 % CISF 2.22– 10.01, Z = 4.03, P = 5.6 × 10–5), CRP*T+IL6*C/G (SF = 2.42, 95 % CISF 1.19–4.94, Z = 2.44, P = 0.015), MSRA*C+CRP*C (SF = 2.56, 95 % CISF 1.12–5.86, Z = 2.22, P = 0.027). Thus, the results suggest that a synergistic effect is observed for these pairs.

Next, to construct a prognostic model for MI, a multifactorial logistic regression analysis was performed with a step-bystep inclusion of the most significant predictors (individual polymorphic variants, as well as significant paired combinations identified during the SF analysis). Table 5 presents the list of predictors included in the final model. Thus, a model for calculating the genetic risk of MI was obtained, which, according to the ROC analysis, has a fairly high prognostic efficiency (AUC = 0.71, PAUC = 1.7 × 10-16, see the Figure).

**Table 5.**
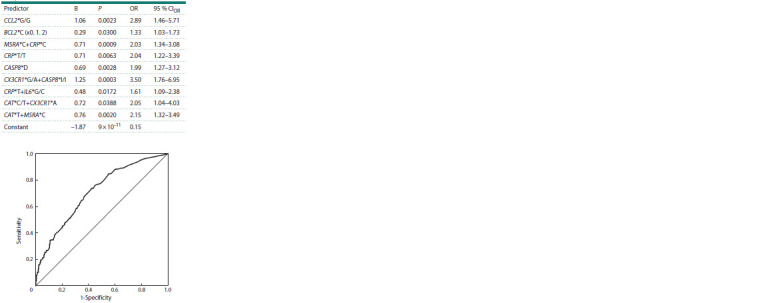
Coefficients of the logistic regression equation for the multifactorial model for calculating the genetic risk of myocardial infarction

## Discussion

The study aimed to identify informationally significant risk predictors of MI. The results indicate the involvement of genes encoding proteins involved in the inflammatory response, antioxidant protection, and apoptosis in forming a predisposition to MI, which is consistent with modern concepts of CHD etiopathogenesis. Indeed, the consequence of oxidative stress is lipid peroxidation and protein oxidation, which are factors of vascular endothelial damage, leading to the activation of inflammatory processes or apoptosis. Moreover, at the early stages of atherogenesis, apoptosis can be considered a protective factor, while at later stages, it is a factor in atherosclerotic plaque destabilization and activates thrombus formation, which is the direct cause of MI.

Fractalkine (CX3CL1) via its receptor (CX3CR1) triggers chemotaxis and monocyte adhesion in the area of atherosclerotic damage at early stages of atherogenesis (Schulz et al., 2007). Also, this chemokine has an anti-apoptotic effect on smooth muscle cells and monocytes and promotes the proliferation and migration of smooth muscle cells, which contributes to the formation and growth of atherosclerotic plaque (Liu et al., 2010). rs3732378 in the CX3CR1 gene determines the replacement of the amino acid threonine with methionine. D.H. McDermott (McDermott et al., 2003) showed that the receptor with 280M (allele *A) binds to fractalkine less effectively, i. e. allele *A was considered as a protective factor in relation to atherosclerosis. At the same time, if at early stages of atherogenesis, the decreased activity of the CX3CL1- CX3CR1 system inhibits the disease development, at later stages, the same effect can lead to apoptosis of monocytes and foam cells, disease progression, and thrombosis (Landsman et al., 2009; Van Vré et al., 2012).

CASP8 belongs to cysteine proteases and triggers a cascade of reactions with the final result of cell apoptosis (Ho, Hawkins, 2005). T. Sun et al. (2007) showed that the deletion of 6 nucleotide pairs in the promoter region of the CASP8 gene (rs3834129) disrupted the binding site for the stimulating protein (sp1) and reduced the transcriptional activity of the gene. In the same work, in vivo experiments demonstrated that the 6N deletion variant was associated with lower apoptotic reactivity of T-lymphocytes when stimulated by cancer cells. Based on this, the identified CX3CR1*G/A+CASP8*I/I variant may be associated with increased apoptotic activity and destabilization of the atherosclerotic plaque. At the same time, for carriers of the rs3834129*D allele, a decrease in apoptotic activity at earlier stages of atherogenesis may contribute to disease progression, which is confirmed by the study of this polymorphic variant in a sample of the Russian ethnic group from Novosibirsk, where an association of the *D/D genotype with progressive atherosclerosis was demonstrated (Maksimov et al., 2022).

Catalase belongs to the group of antioxidant enzymes, catalyzes decomposition of hydrogen peroxide formed in biological oxidation into water and molecular oxygen, and protects cells from damage by free radical oxidation products. H. Yang et al. (2004) demonstrated that mice with ApoE- /- and increased expression of catalase had a slowdown in atherosclerosis development.

Information on the association of the polymorphic variant rs1001179*CAT with enzyme activity is contradictory. Thus, for Americans of European descent, a direct correlation was shown between catalase activity and the allele *C, and the differences in the level of catalase activity varied significantly depending on the level of fruit and vegetable consumption (Ahn et al., 2006); in Italians with chronic lymphocytic leukemia, carriers of the allele *T were characterized by a lower level of methylation and a higher level of the CAT gene expression (Galasso et al., 2022); the work on population samples from Russians and Buryats revealed that carriers of the *T/T genotype had lower concentrations of diene conjugates than carriers of the allele *C, which allows assuming greater catalase activity for individuals with the *T/T genotype (Ershova et al., 2016).

Thus, the CAT*C/T+CX3CR1*A combination identified in the present study may be associated with higher catalase activity, one of the effects of which is proliferation inhibition and induction of apoptosis of vascular smooth muscle cells (Brown et al., 1999) by catalase and a decrease in the inhibitory effect of smooth muscle cells on apoptosis by fractalkine and its receptor.

Methionine sulfoxide reductase A (MSRA) catalyzes the reduction of methionine sulfoxide to the parent methionine. It is believed that a decrease in MSRA activity decreases cellular resistance to oxidative stress. Y. Xu et al. (2020) demonstrated the ability of MSRA to restore the anti-atherogenic function of oxidized high-density lipoproteins. Previously, the authors of the present study found that the *T/T genotype of the rs10098474 polymorphic locus as part of the polymorphic loci combination of the CAT (rs1001179) and GPX1 (rs1050450) genes was more common among individuals over 90 years of age (Erdman et al., 2021), which is consistent with our results on the negative contribution of the allele *C to forming a hereditary predisposition to MI.

According to a number of studies, the *T/T genotype of the rs1205 (CRP) polymorphism is associated with lower plasma CRP levels in Europeans (Kolz et al., 2008), Americans of European descent (Lange et al., 2006), and residents of eastern Mexico (Reynoso-Vilalpando et al., 2021). CRP has pronounced pro-inflammatory effects: according to (Pasceri et al. 2000), it stimulates expression of chemokine intercellular adhesion molecules; H. Fujii et al. (2006) noted that CRP is able to increase the release of reactive oxygen species and induce apoptosis of progenitor endothelial cells, which contributes to endothelial dysfunction.

At the same time, anti-atherogenic properties are also noted – CRP binds modified low-density lipoproteins (Tabuchi et al., 2007); as a result, it can prevent the formation of foam cells and limit complement activation. CRP also inhibits the oxidation of low-density lipoproteins (Badimon et al., 2018). In the model the authors of the present study obtained, the *T/T genotype of the rs1205 polymorphic variant is an MI risk factor, which is consistent with the data on the protective properties of CRP. The authors of the present study also found an unfavorable synergistic interaction in the combination of MSRA*C+CRP*C. Probably, the allele *C rs10098474 of the MSRA gene is associated with decreased enzyme activity and, as a consequence, decreased cellular resistance to oxidative stress, while CRP is known to be able to increase reactive oxygen species release, which can enhance the negative impact of this combination.

The pro-atherogenic role of the chemokine CCL2 (MCP1, a key factor providing chemotaxis of immune competent cells to the site of damage) was demonstrated in the works (Aiello et al., 1999; Öhman et al., 2010). The genotype *G/G rs1024611*CCL2 according to the data (McDermott et al., 2005) is associated with increased CCL2 content in plasma and with MI. The association of the CCL2*G/G genotype with an increased risk of CHD was confirmed by a meta-analysis of European populations, while statistically significant results were not obtained for Asian populations (Bai et al., 2015).

BCL2 is an inhibitor of apoptosis, an intracellular protein, the main representative of the BCL2 family. The C allele of the polymorphic variant rs12454712 is able to bind to the transcription factor ZNF329, which increases the expression of the BCL2 gene (Dong et al., 2021). As already noted, in the later stages of atherosclerosis, activation of apoptosis plays a negative role. At the same time, apoptosis can be a significant factor in limiting intimal hyperplasia in atherosclerosis; in addition, macrophage apoptosis can be a factor in excessive limitation of the inflammatory response (Vladimirskaya et al., 2015).

## Conclusion

Notably, the obtained results can be considered intermediate, since the resulting model has limited predictive ability (which can probably be compensated for by introducing additional predictors). In addition, this model needs to be confirmed on alternative samples. Nevertheless, the results give reason to assume that polymorphic variants rs1205*CRP, rs3732378*CX3CR1, rs1800795*IL6, rs1024611*CCL2, rs3834129*CASP8, rs1042522*TP53, rs12454712*BCL2, rs1001179*CAT, and rs10098474*MSRA significantly contribute to the formation of hereditary predisposition to MI. In addition, it was demonstrated that the identified synergistic interactions between genotypes/alleles in combinations of CX3CR1*A/G and CASP8*I/I, MSRA*C and CRP*C, CAT*C/T and MSRA*C, and CAT*C/T and CX3CR1*A can significantly affect the resulting predictive model. The nature of these interactions is subject for further analysis.

## Conflict of interest

The authors declare no conflict of interest.
